# Barriers and facilitators to the dissemination of national movement behavior guidelines among health-promoting organizations: a qualitative study

**DOI:** 10.3389/fpubh.2024.1470050

**Published:** 2024-12-04

**Authors:** Kaitlyn D. Kauffeldt, Isaac K. McFadyen, Amy E. Latimer-Cheung, Guy Faulkner, Jennifer R. Tomasone

**Affiliations:** ^1^School of Kinesiology and Health Studies, Queen’s University, Kingston, ON, Canada; ^2^School of Kinesiology, University of British Columbia, Vancouver, BC, Canada

**Keywords:** dissemination, 24-hour movement behavior guidelines, complex adaptive organizations, consolidated framework for implementation research, public health

## Abstract

**Background:**

National movement behavior guidelines offer evidence-informed recommendations for how to obtain health benefits. However, their impact on practice and policy has been limited. Factors at multiple levels determine the effective mobilization of knowledge into practice. Historically, little attention has been paid to assessing the social, organizational, and economic factors that influence the uptake of national movement behavior guidelines; potentially contributing to their blunted impact on public health outcomes. The purpose of this study was to explore the barriers and facilitators experienced by intermediary organizations to disseminating national movement behavior guidelines.

**Methods:**

Representatives from organizations involved in the development and dissemination of the Canadian 24-Hour Movement Guidelines for Adults 18–64 Years and Adults 65 Years or Older were invited to participate in semi-structured interviews to explore barriers and facilitators to national movement behavior guideline dissemination. Interview guides were informed by the Consolidated Framework for Implementation Research (CFIR). Fourteen interviews were conducted, and transcripts were analyzed using inductive thematic analysis. Identified barriers and facilitators were mapped onto the CFIR.

**Results:**

Participants identified several elements that have the potential to influence the dissemination of national movement behavior guidelines, such as organizational alignment, resources (i.e., time, human, financial), and ownership of the guidelines.

**Conclusion:**

This study provides insight into the breadth of barriers and facilitators to guideline dissemination that may be experienced by intermediary organizations. Findings may be used to inform interventions designed to improve the dissemination and uptake of national movement behavior guidelines among health-promoting organizations.

## Introduction

The development of national movement behavior guidelines represents a starting point for addressing healthy movement behaviors at a population level (1). Despite their importance, national movement behavior guidelines have had little impact on practice and policy (2–6). Further, the systemic factors that contribute to the poor uptake of national movement behavior guidelines are not well understood (6). One potential explanation for the limited impact of national movement behavior guidelines is the failure of previous guideline development and knowledge mobilization teams to accommodate for the complexity inherent in large-scale knowledge mobilization. For example, previous guideline knowledge mobilization efforts have often relied on single intervention approaches, such as tailoring messages to target audiences and mass media or communications campaigns; with less attention paid to the context and processes through which the knowledge mobilization of movement behavior guidelines occurs (6). Indeed, tailored messages play an essential role in raising awareness and knowledge of national movement behavior guidelines among knowledge users (7, 8). However, an exclusive focus on strengthening the content of guideline messages may continue to have only a partial impact on dissemination outcomes if the myriad of contextual factors that influence successful knowledge mobilization are not considered. Factors at multiple levels (e.g., intrapersonal, interpersonal, organizational, community, and policy) determine the effective mobilization of knowledge into practice (9).

Intermediary organizations (i.e., named for their ‘mediating’ role in moving evidence into practice) are frequently engaged to support the knowledge mobilization of national movement behavior guidelines as they can serve as credible sources of information for knowledge users and are well positioned to reach target audiences. Further, as knowledge mobilization efforts for movement behavior guidelines are often under-resourced (6), engaging intermediary organizations in guideline development and knowledge mobilization can increase the likelihood that guideline dissemination will be meaningfully sustained long-term [i.e., after the funding period has ended (10)]. However, intermediary organizations are complex implementation environments as they are comprised of individuals with their own values, attitudes, and beliefs who form ‘parts’ of organizational structures that respond to internal and external pressures in predictable and unpredictable ways (11). As such, there is merit in exploring the multi-level constructs that may facilitate or impede national movement behavior guideline dissemination at an organizational level.

The Consolidated Framework for Implementation Research [CFIR (9)] is a theory-derived framework that has been widely applied to investigate determinants (e.g., barriers and facilitators) that impact implementation outcomes (12). The CFIR is comprised of 48 constructs and 19 subconstructs that are categorized into five domains. These domains include *innovation* (i.e., the features of innovation, 8 constructs), *outer setting* (i.e., the aspects of the external context or environment, 7 constructs), *inner setting* (i.e., the aspects of the setting in which the innovation is implemented, 11 constructs), *individuals* (i.e., the actions and behaviors of individuals, 13 constructs), and *implementation process* [i.e., the activities and strategies used for implementation, 9 constructs (9)]. The CFIR is well-suited to articulating the multi-level determinants to organizational dissemination, as it implies a systems approach to understanding the influences on knowledge mobilization [i.e., looking ‘upwards’ and ‘downwards’ to the complex array of factors that influence knowledge mobilization outcomes (9)]. Accordingly, the purpose of this study was to apply the CFIR to explore the barriers and facilitators experienced by intermediary organizations to disseminating national movement behavior guidelines. Findings may yield novel insights into the systemic factors that influence the dissemination of national movement behavior guidelines at an organizational level and provide improved guidance to guideline developers and knowledge mobilization teams on how to mobilize guidelines into practice.

## Methods and methodology

### Context

In October 2020, the Canadian 24-Hour Movement Guidelines for Adults Aged 18–64 Years and Adults Aged 65 Years or Older [24HMG (13)] were released, thereby completing the full suite of national movement behavior guidelines for individuals of all ages in Canada. The development and release of the 24HMG represented a timely opportunity to explore the multi-level barriers and facilitators experienced by intermediary organizations to national movement behavior guideline dissemination.

### Philosophical assumptions

We approached our work from a critical realist paradigm, as it allows for insights into the barriers and facilitators experienced by intermediary organizations to disseminating national movement behavior guidelines while situating our findings within the broader context for dissemination (i.e., the system level or emergent influences on dissemination). As such, this study is grounded ontologically in realism and epistemologically in constructivism/interpretivism (14). Realist ontologies assume that there is an independent reality or ‘truth’ that can be known while acknowledging that it may never be fully understood, as systems interact in ways that can create unpredictability and uncertainty. Constructivist/interpretivist epistemologies are premised on the belief that knowledge is co-constructed (e.g., between researchers and participants) and situated within a social context (15). Therefore, it is necessary to state the position and background of the research team conducting this work and their potential influence on the research process. At the time of data collection, the first author was a doctoral student in the field of health promotion. The first author has a background in implementation science and played a substantial role in completing the formative research for the knowledge mobilization of 24HMG. The first author recognizes that their background may have influenced data collection and analysis in various ways. For example, it is possible that their theoretical background and knowledge of implementation science shaped the organizational barriers and facilitators that were described in this study. In addition, their access to study participants was aided by their previous experience completing formative research for the 24HMG. Next, several members of the research team (JRT, ALC, GF) held leadership positions on the Knowledge Mobilization Advisory Committee for the 24HMG, a committee responsible for conceptualizing and implementing the knowledge mobilization approach for the 24HMG. These researchers are university professors and are experts in fields of knowledge mobilization and qualitative research. Each contributed meaningfully to the design of this study and the interpretation of results. Given the roles and positions of our research team, it is possible that participants were less forthcoming about any pressures or challenges experienced by their organization to disseminating the 24HMG. Further, it is also possible that participants may have been more motivated to positively frame their dissemination plans for the 24HMG.

### Participants

Following institutional ethics approval, members belonging to organizations involved in the development (13) and knowledge mobilization (16) of the 24HMG were invited to participate in semi-structured interviews via email in June 2020 (4 months before the launch date of the 24HMG^1^). Note that in cases where more than one member from an organization was involved in the 24HMG initiative, an email was sent to each representative asking that they collectively nominate one individual who was well suited to complete the interview, or they were offered the opportunity to participate in data collection together.

### Measures and procedure

Prior to the interview, participants were sent a brief online survey to collect demographic information about themselves (e.g., history with organization, position) and their organization (e.g., location, level, sector; Supplementary material S1). The interview guide was developed in collaboration with members of the Knowledge Mobilization Advisory Committee for the 24HMG (16) and structured using the CFIR^2^ (17) (Supplementary material S2). The guide was pilot tested with members of the research team to assess the meaningfulness and clarity of interview questions. All interviews were conducted by the first author using Zoom. To stimulate discussion, pre-interview materials containing strategies used for previous national-level movement behavior guideline dissemination and methods for their evaluation (6) were distributed to participants prior to the interview (Supplementary material S3). Two interviews were completed with two participants and 12 interviews were completed with one participant. Interviews lasted between 38 and 69 min (*M* = 55.9) and were audio recorded and transcribed verbatim. Field notes were kept by the first author throughout the interview process. Interview participants were offered a $50 CAD e-gift card in compensation for their participation.

### Data analysis

Descriptive statistics were calculated for demographic data. A summary of characteristics for participating organizations can be found in Table 1. To protect confidentiality, organizations were numbered, and participants were assigned pseudonyms. Qualitative analysis followed the six-stage process as outlined by Braun and Clarke (18). Recently, there has been increased dialogue regarding the misapplication of thematic analysis within the domain of qualitative research (19). With knowledge of these criticisms, we chose to use inductive thematic analysis as it can be used flexibly with a range of research paradigms [i.e., critical realism (20)] and it allows for the development of cohesive themes across complex data sets. Further, the use of inductive thematic analysis allows for the identification of a breadth of determinants, increasing the likelihood of identifying important themes that may not be effectively captured by the CFIR (12). First, the first author familiarized herself with the data through listening to audio files and re-reading interview transcripts. During this stage the first author recorded any interesting features or patterns within the data. Next, the transcribed data was transferred into a qualitative analysis software [NVivo Version 12 (21)] and the salient features of the data were systematically labelled and organized into initial codes. Initial codes were then sorted and organized into candidate subthemes and themes. During this stage, the first author engaged in discussions with a research assistant (IKM) who independently reviewed and coded a sample of the transcripts to stimulate further discussion and reflection of the generated codes or candidate subthemes and themes. Codes and themes were revised based on discussion. It was during this step that the CFIR (9) was deductively applied. We chose to use the updated CFIR (9) to meaningfully situate our findings in current implementation science literature. Finally, all codes and themes were reviewed by the first author and cross referenced across the entire data set to develop a robust understanding of the barriers and facilitators experienced by intermediary organizations to disseminating national movement behavior guidelines. Throughout the analytic process, the first author engaged in iterative discussions with a critical friend (i.e., ‘a theoretical sounding board’; JRT) to explore alternative interpretations of the data and encourage reflexivity (22).

**Table 1:** Characteristics of participating organizations.

Characteristic	Frequency (%)
Province	
Alberta	1(7.14)
British Columbia	2(14.29)
Ontario	10(71.43)
Nova Scotia	1(7.14)
Level	
National	6(42.86)
Provincial/Territorial	6(42.86)
Local	2(14.29)
Sector	
Government	2(14.29)
Not-for-profit	7(50.00)
Private	0(0)
Education	5(35.71)
Number of full-time employees	
<10	4(28.57)
10–19	2(14.29)
20–29	1(7.14)
30–39	0(0)
40+	3(21.43)
Not collected^a^	4(28.57)
Number of part time employees	
<10	7(50.00)
10–19	0(0)
20–29	0(0)
30–39	0(0)
40+	3(21.43)
Not collected^a^	4(28.57)
Volunteers	
<10	5(35.71)
10–19	2(14.29)
20–29	0(0)
30–39	0(0)
40+	3(21.43)
Not collected^a^	4(28.57)
History of movement behavior promotion	
<5 years	1(7.14)
5–10 years	3(21.43)
11–15 years	4(28.57)
16–20 years	1(7.14)
20+ years	5(35.71)
Movement behaviors promoted	
Physical activity	13(92.86)
Sedentary behavior	9(64.29)
Sleep	4(28.57)
Percentage of resources allocated to movement behavior promotion	
<20	5(35.71)
20–39%	0(0)
40–59%	0(0)
60–79%	1(7.14)
80–100%	4(28.57)
Not collected^a^	4(28.57)
Previous movement behavior guideline dissemination	
Physical activity	13(92.86)
Sedentary behavior	6(42.86)
Sleep	2(14.29)
None	1(7.14)

In cases where *n* > 14, participants selected multiple responses for their organization.

a Content experts were unable to answer item on behalf of their organization.

### Methodological rigor

As interpretations of rigor within qualitative research can vary amongst scholars, researchers are encouraged to demonstrate rigor using the criteria that are most aligned with the philosophical assumptions embodied within their work (23). As critical realism acknowledges that our identities and experiences are intertwined with the knowledge we create, reflexivity was used as both a strategy and a marker of quality throughout this research. The inclusion of a reflexive statement on philosophical assumptions and positionality demonstrates how our backgrounds and theoretical perspectives shaped the study’s design, analysis, and reporting. Further, the first author engaged in discussions with an independent coder and a critical friend (22) throughout data analysis, prompting her to critically reflect on her expectations for intermediary organizations to disseminate guidelines and consider alternative interpretations of participant data. Next, it was important to ensure that the results of this study were credible and generalizable to our audiences (guideline developers, health-promoting organizations). These criteria were achieved by exploring multiple perspectives of organizational barriers and facilitators to guideline dissemination. Further, rich descriptions and participant quotes were used to encourage readers to interpret the results of this study and draw their own conclusions based on context. Lastly, semi-structured interviews were used to maintain coherence between our philosophical assumptions and research methods. Interviews facilitated the exploration of participants’ experiences while also seeking to uncover the underlying structures that influence their actions and perspectives.

## Results

Participants identified several elements that have the potential to influence the dissemination of national movement behavior guidelines, with some elements reported as either a barrier or a facilitator according to the dissemination context. Barriers and facilitators influencing guideline dissemination were grouped into four overarching themes—*Compatibility*, *Capacity*, *Actions*, and *Conditions*, with a total of 20 subthemes. For brevity and clarity, the following section provides a high-level overview of the resulting themes and subthemes. Findings are reported as both barriers and facilitators unless otherwise stated.

### Compatibility

The compatibility or ‘fit’ of guideline dissemination with an organization was reported by participants as being highly important for dissemination. Several aspects of compatibility were described, including having an ‘institutional memory’ for dissemination, alignment with an organization’s purpose and/or processes, and alignment with an individual’s role or job description.

#### ‘Institutional memory’ for dissemination

Participants described that having prior related experience disseminating national guidelines at an organizational level was important for future guideline dissemination. In many cases, institutional memory that facilitated dissemination was linked to an organization’s involvement in previous guideline dissemination initiatives. When there was no precedent for guideline dissemination, participants reported having low confidence or feeling uncertain about how their organization would disseminate guidelines. As one participant explained:

I guess thinking back to the fact that we have never disseminated any guidelines for any organization, so there is no precedent for us to go off of, of what we have never done before, right? … So, I guess there would be a little bit of that learning to be done there, or seeing what we are comfortable with doing (Melissa, Organization 3).

#### Alignment

Various forms of organizational alignment with guideline dissemination were described by participants. The most frequently discussed form of alignment was the degree to which guideline dissemination aligned with the purpose or goals of an organization (i.e., ‘alignment with mission’). In many cases, participants seemed to view this form of alignment as a pre-requisite for dissemination, wherein the degree of alignment was described as proportional to an organization’s commitment to—and investment in—dissemination. As one participant noted:

It is like if we were to disseminate fall guidelines for older adults, I mean that is not our primary group of interest, right? And so, there is a bit of a mismatch, people aren’t going to look to us for that and so we are not going to put in the same effort as we did for the previous guidelines because it is not our thing (John, Organization 5).

The second form of alignment described by participants was the alignment of guideline dissemination with an organization’s processes or day-to-day operations. In this case, the degree of alignment was described as related to the feasibility or efficiency of guideline dissemination (e.g., the amount of resources that would need to be reallocated for successful dissemination). As one participant described:

We’ve [disseminated] for all kinds of other things, this fall we would be disseminating our physical activity results … it is fairly easy for us to get the information out to our database, so I am not concerned that we would not be able to do it (Ellen, Organization 7).

The last form of alignment described by participants was the extent to which guideline dissemination aligned with their role or job description, wherein the degree of alignment was connected to the responsibility participants felt to disseminate the guidelines. As one participant noted, “[disseminating guidelines] is just my job, I think it connects very well to my responsibilities … I do not see it as an add on, I see it as a reasonable expectation” (Cynthia, Organization 1).

### Capacity

The impact of organizational capacity on guideline dissemination was echoed among all participants. The capability of an organization to disseminate guidelines was described as closely tied to the following: the presence of siloed systems, resources for dissemination, competing priorities, decision-making authority, and individual knowledge and experience.

#### Siloed systems (barrier only)

A number of participants believed that an organization’s structure presented barriers to guideline dissemination. Specifically, participants highlighted that the presence of siloed systems within an organization had the potential to disrupt the process of dissemination due to communication breakdown or inefficient collaboration across departments. One participant described this challenge as follows, “we can brief up, we can let people know that [the guidelines] are out, but from each of us in our own silos within the organization, we are all going to do something different right?” (Megan, Organization 9). This barrier was particularly salient for participants representing large organizations operating on a national level and academic institutions.

#### Resources for dissemination

Three types of resources were reported as essential for guideline dissemination. First, financial resources were described as having an important influence on the quantity and quality of dissemination that could be performed by organizations. Several participants felt that their organization did not have the funds needed to meaningfully disseminate guidelines (e.g., use an approach that would result in substantial changes in awareness and knowledge of guideline recommendations at a population level). As one participant described:

I mean if we produced [the guidelines] and did not disseminate them, then they have zero chance. So, it has to be the start point, unfortunately, and this is where the resources come in, it becomes the endpoint as well (John, Organization 5).

In the absence of sufficient funds, many participants described planning for strategies that required fewer resources, such as email distribution, website content creation, or posts on social media. Next, participants agreed that the presence of a dedicated team (i.e., ‘human resources’) was important for supporting guideline dissemination priorities. Participants representing organizations with the presence of collaborative, interdisciplinary teams for communication described a greater capacity for disseminating guidelines. As one participant highlighted, “for us [dissemination] is really important, and in that sense, because we are smaller, we have the benefit of being more flexible and nimble, and we are able to respond pretty quickly” (Elizabeth, Organization 8). Lastly, all participants expressed a preference for materials to support guideline dissemination. Participants felt that having access to packaged, tailored materials for guideline dissemination would ensure consistent messaging of the guidelines to target audiences and enhance the saliency of guideline messages to their professional network. Participants also suggested that having access to materials to support guideline dissemination may resolve some capacity-related concerns to disseminating guidelines. As one participant explained:

The way that our jobs typically work, there are tons of ebbs and flows, so I think having something where if you do have a pocket of time, it’s really easy to push out [would be good] versus something that has fallen down your priority list several times (Krista, Organization 10).

#### Competing priorities

The influence of competing priorities on guideline dissemination was expressed by all participants. In most cases, participants felt that the sheer number of other higher priority organizational initiatives acted as a barrier to both the time and resources that could be allocated to support guideline dissemination. One participant described this challenge as follows:

I think our priority would have to be on getting the things done that we have on the go, we do want to be a good partner and share as much as we can, but in terms of devoting our own resources for [dissemination] … people’s hands are a little bit tied up right now (Melissa, Organization 3).

Notably, only one participant reported the absence of competing priorities as a facilitator.

#### Decision-making authority

Having the authority to influence organizational directives was highlighted by several participants as an important determinant of guideline dissemination. When reported as a barrier, participants felt that they had a low level of decision-making authority or agency within their organization and that higher levels of approval were needed to disseminate the guidelines. As one participant explained:

I personally have like this much control, itty bitty control over where they get disseminated and who they get disseminated to. I have put a suggestion to my manager … and it’s up to my manager to decide whether or not she approves it (Megan, Organization 9).

In contrast, participants who described decision-making authority as a facilitator of dissemination often held positions or roles where they could directly influence organizational priorities (e.g., directors or members of smaller organizations with a ‘flat’ organizational structure).

#### Knowledge and experience

Two categories of knowledge and experience were described by participants as playing an important role in guideline dissemination. First, participants felt that a level of expertise in knowledge mobilization was needed to effectively disseminate the guidelines (i.e., ‘dissemination knowledge and experience’), specifically in regard to the messaging of guideline content. When reported as a barrier, participants felt that they did not possess the expertise needed to craft guideline messages that would resonate with their organization’s target audience. As one participant described, “quite frankly, this is not my primary area of research, I would want to do the message, I would want to be faithful to the message” (Lauren, Organization 6). Next, a few participants also explained that an understanding of guideline content (e.g., the compositional nature of movement behaviors) and its relevance may be helpful for supporting the dissemination of movement behavior guidelines (i.e., ‘knowledge and understanding of guideline content’). When described as a barrier, participants suggested that the integration of movement behaviors (i.e., physical activity, sedentary behavior, sleep) may not be a concept that is easily understood among members of their organization, and as a result, it may decrease motivation to disseminate the guidelines or increase the prioritization of select movement behaviors (e.g., only physical activity) in guideline messages. For example, one participant described:

It’s all about having them understand what their role is in relation to the full guidelines … in sport it has been a challenge to get them to think about sedentary behavior ‘cause they just want to focus on moderate to vigorous [physical activity] (Cynthia, Organization 1).

### Actions

Participants believed that the strategic actions or decisions of an organization have an impact on successful guideline dissemination. These decisions include utilizing planned activities for dissemination, appointing an internal champion, anticipating dissemination outcomes, and leadership buy-in for dissemination.

#### Utilizing planned activities for dissemination (facilitator only)

Participants described that embedding guideline content into planned or existing activities was a resource (i.e., financial, human, time) efficient strategy for facilitating guideline dissemination, as guideline content could be easily integrated into related organizational initiatives. As one participant commented, “I have always been a fan of working within pre-existing structures … having opportunities to disseminate in unique places is always great, but often a lot more work” (Krista, Organization 10). For example, several participants suggested integrating messages about the guidelines into their organization’s social media campaign while others discussed the potential to share information about the guidelines at regularly scheduled hospital ‘lunch and learns’.

#### Internal champion (facilitator only)

A few participants felt that having an internal champion who was committed to guideline dissemination was essential for overcoming the internal barriers experienced when moving dissemination initiatives forward within an organization. As one participant noted, “you need an internal influencer, someone who is willing to take up the banner and move bits forward” (Martin, Organization 2). Internal champions were described as influential leaders within an organization with a vested, intrinsically motivated, interest in promoting guidelines to target audiences. This facilitator was most frequently mentioned by participants representing complex implementation environments, such as large national organizations or academic institutions.

#### Anticipated dissemination outcomes (facilitator only)

The ‘knowable’ outcomes of guideline dissemination were described by a minority of participants as an incentive for their organization to disseminate the guidelines. Various benefits to guideline dissemination were described by participants. For example, one participant suggested that disseminating the guidelines would “reinforce our role as a provider of knowledge to our membership and outreach groups” (Martin, Organization 2). Similarly, another participant expressed that disseminating guidelines would strengthen their existing partnerships within their professional network and grow the network of their organization. These anticipated outcomes were perceived to work collectively to drive the strategic planning initiatives of an organization.

#### Leadership buy-in for dissemination

Many participants described that having a commitment to dissemination among organizational leadership was essential for successful guideline dissemination. When discussed as a facilitator, participants described that the top-down commitment from organizational leadership increased the likelihood that dissemination would be prioritized (e.g., as an internal directive) and the potential for time and resources to be reallocated to improve the quantity and quality of dissemination. When described as a barrier, participants reported experiencing a lack of accountability for dissemination, viewing guideline dissemination as a task that is performed because of personal interest, rather than as a task mandated by their organization. For example, one participant explained, “besides my own desire to see, to do the best work, this is not something my supervisor is like ‘you have to do’, like there is not a lot of accountability unfortunately” (Krista, Organization 10).

### Conditions

Lastly, participants described several aspects of the dissemination climate (e.g., internal or external to their organization) that have the potential to influence guideline dissemination. These aspects include the frequent turnover of guidelines, COVID-19 related factors, partnerships and connections, external pressure to disseminate, positive attitudes towards the guidelines, the socio-political context, organizational culture, organization readiness to disseminate, and a sense of ownership of the guidelines.

#### Frequent turnover of guidelines (barrier only)

A small number of participants suggested that the frequent turnover of movement behavior guidelines in Canada may act as a deterrent to organizational ‘buy-in’ to guideline dissemination. As one participant noted, “we have conditioned all of our partners that [the guidelines] will change constantly and we will call on something different, the promotion will stop and start and so no one really buys in” (John, Organization 5). Specifically, participants felt that organizations with a history of involvement in guideline dissemination initiatives may be less motivated to pool their resources to implement the knowledge mobilization plan for the newest version of movement behavior guidelines, as there is a high likelihood that it will not be sustained over time.

#### COVID-19 related factors (barrier only)

Participants described several challenges to guideline dissemination that were experienced as a direct result of the COVID-19 pandemic. These barriers worked conjointly to reduce the organizational capacity for guideline dissemination and included having a decreased priority for guideline dissemination (i.e., competing priorities), experiencing constraints to traditional forms of dissemination (e.g., in-person seminars, workshops, conferences), and experiencing technological difficulties to virtual forms of dissemination. Of these barriers, the most frequently described barrier was the presence of competing priorities. Participants felt that they could not commit their organization to guideline dissemination, as many of their resources (i.e., financial, human, and time) were allocated to disseminating information about the COVID-19 pandemic to their target audience. As one participant expressed:

It is a workload issue frankly. COVID-19 has put a lot of pressure on our organization as a whole. We have a big communications department and 99% of them are working on COVID, and 1% of them are working on everything else (Megan, Organization 9).

#### Partnerships and connections (facilitator only)

The cultivation of trusted partnerships and connections was described by all participants as a valuable facilitator of guideline dissemination. Participants agreed that having an established network for dissemination was important for increasing the reach of guideline materials and messages to receptive target audiences. As one participant described, “from our perspective [dissemination] is easy, because we already have that pre-set group of people who are interested” (Ellen, Organization 7). Another participant described dissemination as a game of telephone, suggesting that the maintenance of strong partnerships was essential for the communication of guidelines. A second advantage to cultivating strong partnerships described by participants were the opportunities that were provided to learn (i.e., ‘peer learning’) from other organizations operating in similar dissemination and/or implementation contexts. Many participants described experiencing unique barriers or facilitators to dissemination and valued having opportunities to share health-promoting approaches with other like-minded organizations with similar organizational structures.

#### External pressure to disseminate (facilitator only)

One participant reported that a moderate amount of social pressure has the potential to act as a catalyst for participation in guideline dissemination. Although this facilitator was not described by many participants, this participant expressed that experiencing social pressure for their organization to be involved in the guideline development and dissemination process encouraged their organization to subsequently renew their commitment to guideline dissemination, expressing “and here we are again, proud to be part of the whole process” (Megan, Organization 9).

#### Positive attitudes towards the guidelines (facilitator only)

A few participants described that having positive attitudes towards guideline content and materials increased their motivation to engage in guideline dissemination at an individual level. As one participant stated, “I am confident that they will be disseminated … everyone is excited about the launch and it is seen as important” (Ellen, Organization 7). Participants felt that the 24HMG were high-quality, credible (i.e., evidence-informed), and relevant, and described feeling an increased responsibility or stewardship to disseminate them to their target audience.

#### Socio-political climate

The social or political narratives in Western culture were discussed by participants as being a strong driver of organizational priorities in ways that could support or hinder guideline dissemination. For example, when described as a facilitator, participants recounted their experience working within an environment that was ‘resistant’ to the implementation of a health-promoting initiative, until the health topic gained traction in the media cycle (i.e., social media, news outlets, radio) which quickly shifted the priorities of their organization. As one participant described:

I have definitely been in meetings where I walk in and realize that the [news] story I heard this morning on the way is going to completely change the conversation that comes up, even though I spent 20 h putting this presentation together, the only thing they are going to ask me about is the one little thing that they happened to hear on the drive in (Krista, Organization 10).

#### Organizational culture

A small number of participants expressed that organizational culture has the potential to influence the feasibility and efficiency of guideline dissemination. When viewed as a facilitator, participants described that their organization embodies a culture of wellness at all levels, working synergistically to support the dissemination of movement behavior guidelines both within the organization and to target audiences. In the absence of such a culture, participants described relying on individualized or grassroots efforts to counter the ‘resistance’ they experience to guideline dissemination within their organization. As one participant explained, “I think we are kind of at the grassroots level, and we can work together on it, but certainly budgets and structure does not support it” (Chris, Organization 13).

#### Organization readiness to disseminate

Participants agreed that organizational readiness to disseminate guidelines plays an important role in guideline dissemination. Participants described readiness as having advanced notice of when the guidelines would be released and having time to plan and prepare their dissemination approach. Interestingly, participants felt that being engaged in the process of guideline development and dissemination improved their sense of readiness to disseminate guidelines, as it provided them with ‘designated’ time to think about dissemination. When reported as a barrier, participants represented organizations (e.g., academic institutions) that planned their health-promoting activities 1 year in advance. In these cases, participants expressed that the timely dissemination of guidelines would be challenging, as their resources were already allocated to other initiatives. As one participant explained:

We will not be able to turn these things around in a week. We typically take months and months to plan something out and so, the earlier that people can know that something is coming down the pipe, or opportunities to engage, the more effective (Owen, Organization 12).

#### Ownership of the guidelines

Having a sense of ownership of the guidelines was reported by all participants as essential for investing in guideline dissemination. When described as a barrier, participants viewed the guidelines as external resources that their organization was requested to promote. In this case, participants did not feel a need to redirect their organization’s resources (i.e., financial, human, and time) to support dissemination, as their organization did not receive funding to develop or disseminate the guidelines. As one participant expressed, “it is basically what is funded and what is not funded, and because you are the guys with all the funding, we figure you better do the work because we ain’t got the cash to pay for it” (Martin, Organization 2). When described as a facilitator, participants expressed feeling a responsibility to disseminate the guidelines, as their organizations have a vested interest in disseminating them given their involvement in guideline development/knowledge mobilization, with or without direct funding for guideline dissemination.

### Mapping to the CFIR

Mapping of the barriers and facilitators to relevant CFIR domains (9) along with illustrative quotes can be found in Table 2. Barriers and facilitators were identified across all CFIR domains, including the *innovation* (*n* = 1), *outer setting* (*n* = 5), *inner setting* (*n* = 13), *individuals* (*n* = 6), and *implementation process* (*n* = 1) domains. However, the majority of determinants were mapped to the inner setting, individuals, and outer setting domain.

**Table 2:** Themes, subthemes, and CFIR domains.

Subtheme(s)	Barrier/facilitator	Definition	Representative quote	CFIR domain
*Theme 1: Compatibility*	*The compatibility or fit with an organization’s purpose and processes*			
‘Institutional memory’ for dissemination^c^		History of movement behavior guideline dissemination within an organization (i.e., whether there is a precedent established for ‘how’ to disseminate guidelines).	“I guess thinking back to the fact that we have never disseminated any guidelines for any organization, so there is no precedent for us to go off of, of what we have never done before, right? … so I guess there would be a little bit of that learning to be done there, or seeing what we are comfortable with doing.”—Melissa, Organization 3 “I mean, it’s our third kick at the can, and so I think we have learned a lot in terms of what worked well [within our organization] and what did not. We have that kind of luxury in learning from past efforts.”—Elizabeth, Organization 8	IS
Alignment^c^	Alignment with mission	Alignment of guideline dissemination with the mission and mandate of an organization	“The fact is, this [guideline] is for adults, so we are not going to be as proactive as we would be, because it is not our primary group of interest.”—John, Organization 5 “I would not be engaged if there wasn’t close alignment with the work we are doing”—Martin, Organization 2	IS
	Alignment with processes	Alignment of guideline dissemination with the information sharing systems and processes of an organization.	“We just generally, we have the infrastructure to disseminate”—Esther, Organization 4 “We know that dissemination is feasible, this is what we routinely do, and we have had success with it”—John, Organization 5	IS
	Alignment with individual role	Alignment of guideline dissemination with the role of the individual who will be disseminating the guidelines.	“It’s my job, [dissemination] connects very well to my responsibilities”—Cynthia, Organization 1	I
*Theme 2: Capacity*	*The capability of an organization to disseminate the guidelines*			
Siloed systems^a^		The presence of siloed systems within an organization can disrupt communication processes and impede guideline dissemination.	“As I said, we can brief up, we can let people know that the guidelines are out there, but from each of us in our own silos within our organization we are going to do something different.”—Megan, Organization 9 “It’s complicated, it’s government, I think you are going to get that no matter what government you are dealing with. You deal with levels of approvals and you deal with various centers that are focused on their own work.”—Megan, Organization 9	IS
Resources for dissemination^c^	Financial resources for dissemination	The financial resources available to support guideline dissemination.	“Certainly in terms of funding and staff and so on it’s always shoestrings, shoestrings, shoestrings to be able to disseminate as widely as we’d like to.”—Esther, Organization 4 “You’re gonna do it for one year and its going to go away, it’s a waste of time, it’s a waste of time in my view because we gotta buy in for the long haul and for that we need [funding].”—John, Organization 5	IS
	Human resources for dissemination	The human resources available to work collaboratively to support guideline dissemination.	“It’s literally time and capacity at the moment, we are already working with you know, somebody only at half time … it sort of becomes that prioritization of work, trying to figure out what are the biggest things that we are getting asked to do.”—Krista, Organization 10 “Previously I would have said yes, but with this new health promotions coordinator that we have, like even materials that go out, we previously had to outsource that kind of stuff, we did not have the capability in house … but now we do, so I think we are definitely gaining that capacity that we did not have before.”—Melissa, Organization 3	IS
	Materials for dissemination	Access to tailored content (e.g., messages, infographics, email scripts) to support guideline dissemination.	“If we are talking about passive dissemination, its, it would not take much of a backseat, but certainly any sort of active form, yeah it would definitely take sort of a backseat to the other priorities that are going on right now, and the deadlines that we have to meet ourselves”—Melissa, Organization 3 “I would really like it if someone else created [tailored materials] it’s just easier, and it’s more likely that it’ll be effective, because if its created at a centralized place for organizations across Canada, it’ll probably be done with a different level of professionalism and strength related to communication.”—Cynthia, Organization 1	IS
Competing priorities^c^		The presence of other organizational initiatives take priority over disseminating the guidelines.	“I would say we are somewhat selfish as an organization, I think our priority would have to be on getting things done that we have got on the go, we do want to be a good partner and share as much as we can, but in terms of devoting our own resources, peoples hands are a little tied up right now.”—Melissa, Organization 3 “And because we do not have competing things, it is easier for us to put focus on [the guidelines], really promote it, do it in a number of different ways at different times.”—Ellen, Organization 7	IS
Decision-making authority^c^		Whether individuals who will be disseminating the guidelines have the agency within their organization to guarantee that guideline dissemination will occur.	“I wish I could commit us to more active [dissemination] but that is beyond my decision-making level and I cannot commit us.”—Melissa, Organization 3 “I have complete freedom to do what I think we should as an organization … I think it is an important piece of work and so we should be involved in it.”—Ellen, Organization 7	I
Knowledge and experience^c^	Dissemination knowledge and experience	Whether individuals who will be disseminating the guidelines have the scientific knowledge or experience to tailor guideline content and materials to target audiences.	“Quite frankly, this is not my primary area of research, I would want to do the message, I would want to be faithful to the message, but … I do not think we can underestimate the science that goes behind strong messaging and communication.”—Lauren, Organization 6 “It’s having the knowledge of how to pull pieces of information out and put that into bit-sized pieces that catch people’s attention … we are fairly adept at pulling out that key information that will grab people’s attention and then be shared.”—Ellen, Organization 7	I
	Knowledge and understanding of guideline content	Whether individuals who will be disseminating the guidelines have an understanding of the guideline content (i.e., movement behavior) and its relevance.	“Often times it comes down to a table of people making decisions on topics that they do not have an extensive evidence-based background in … that is the nature of working in an institution where you have health decisions that aren’t made by health people.”—Krista, Organization 10 “The more comfortable I feel about the information, the easier you make it for me to understand the information, the more I’m gonna be apt to bring it up in the opportunities that I have.”—Chris, Organization 13	I
*Theme 3: Actions*	*The actions or decisions that influence guideline dissemination*			
Utilizing planned activities for dissemination^b^		Organization activities are strategically adapted for guideline dissemination.	“I have always been a big fan of working within pre-existing structures, like over the times, you know having opportunities to disseminate in unique places is always great, but often a lot more work so, if there’s already structures of mechanisms that we can use it is easier.”—Krista, Organization 10	IM
Internal champion^b^		An individual within an organization who is willing to ‘champion’ the dissemination of the guidelines and move dissemination initiatives forward.	“Sometimes I think that if there is not a champion in an organization, nobody will be the champion in the organization. I think you need someone that sees the value, sees it as part of their role and then pulls in all the other groups.”—Chris, Organization 13 “You need an internal influencer, someone who is willing to take up the banner and move it forward.”—Martin, Organization 2	I
Anticipated dissemination outcomes^b^		Anticipated outcomes act as an incentive to disseminate the guidelines (e.g., reinforced ‘status’ within professional network, strengthened relationships with partner organizations).	“So [disseminating the guidelines] gives us that opportunity to maybe further connect with or build stronger rapports with organizations or individuals that we might not have otherwise come into contact with right away. So I think that’s an important one.”—Elizabeth, Organization 8	IS
Leadership buy-in for dissemination^c^		Extent of commitment from organization leadership to prioritize guideline dissemination.	“The approval is there from the top down, so I think if everyone is sort of, on the lower end of the ladder was all for it, but the higher end was opposed I could see it [being a barrier].”—Melissa, Organization 3 “Our organization is lucky that we had multiple senior leaders at the same time say this is really important and built a 20 year history of [health promotion] with sustainability.”—Owen, Organization 12	IS
*Theme 4: Conditions*	*The climate (i.e., internal or external) or context for guideline dissemination*			
Frequent turnover of guidelines^a^		The frequent turnover of movement behavior guidelines negatively influences organizational buy-in.	“The other thing is that we have conditioned all of our partners that [the guidelines] will change constantly and we will call on something different, the promotion will stop and start and so no one really buys in, like they buy in overall, but to the latest gimmicky little program, they do not dig in because they know it is not going to be there tomorrow.”—John, Organization 5	IN
COVID-19 related factors^a^		Challenges to guideline dissemination that are specific to the COVID-19 pandemic.	“If we are in the middle of wave two and the numbers were huge again, then I think you would see less likelihood of getting on the dance card … but it would happen eventually.”—Martin, Organization 2 “What led us to talk about these dissemination strategies was the absence, due to covid, the absence of being able to present the work in the scheduled symposia.”—Esther, Organization 4 “I mean one barrier that seems to be coming up for a lot of people right now, we all are working remotely, and we do not have those, like natural conversations where you are walking from a meeting or to a meeting … you have to be much more deliberate you know?”—Krista, Organization 10	O
Partnerships and connections^b^	Established dissemination network	The extent to which an organization maintains trusted partnerships and a professional network for guideline dissemination.	“We know that it is feasible because there are established paths of communication and there are people who are invested in it.”—Margaret, Organization 14	O
	Peer learning	An organization has the ability to learn from the successful dissemination practices of other similar organizations within their network.	“It’s really been more about how do we connect the dots within and across universities and have more conversations in this space, and then it is pretty remarkable to see how momentum will continue to build once we just create this composition [of institutions].”—Owen, Organization 12	O
External pressure to disseminate^b^		Pressure from other individuals or organizations to disseminate the guidelines.	“You know, quite frankly there was pressure for us to be involved, and they are right, because we do have a vested interest in them and we have been, so we are here again and proud to be a part of the whole process for the guidelines.”—Megan, Organization 9	O
Positive attitudes towards the guidelines^b^		Individual(s) who will be disseminating the guidelines are intrinsically motivated.	“I am confident that they will be disseminated. … everyone is excited about the launch and it is seen as important”—Ellen, Organization 7	I
Socio-political climate^c^		The extent to which the social or political narratives that dominate the public influence organizational priorities for dissemination.	“I have definitely been in meetings where I walk in and realize that the [news] story that I heard this morning on the way in is going to completely change the conversation that comes up, even though I spent, you know 20 h putting this presentation together, the only thing they are going to ask me about is this one little thing that they happened to hear on the drive in.”—Krista, Organization 10 “I think there will be an interest to [disseminate the guidelines] I see a relevance to physical health to mental well-being especially in this current circumstance where students are feeling really isolated.”—Margaret, Organization 14	O
Organizational culture^c^		The extent to which the shared belief, values, and norms within an organization influence guideline dissemination.	“I think there is a big recognition that you know, to create a culture of wellness on campus, you have to hit all of the people that actually come to campus, the whole community … I think we are kind of at the grassroots level, and we can work together on it, but certainly budgets and structure does not support it.”—Chris, Organization 13 “I think then too, if you create a shared vision, I mean a shared acknowledgement of the importance you know, you’ll have way more success too.”—Chris, Organization 13	IS
Organization readiness to disseminate^c^		The extent to which an organization is ready to disseminate the guidelines (e.g., organizations have been primed and have planned for guideline dissemination).	“You do not have the guidelines until you have the guidelines right? We thought that we were going to have certain materials much earlier than we did, we planned for that … you know even a few months makes a difference.”—Esther, Organization 4 “We will not be able to turn these things around in a week. We typically take months and months to plan something out and so, the earlier that people can know that something is coming down the pipe, or opportunities to engage, the more effective.”—Owen, Organization 12	IS
Ownership of the guidelines^c^		The extent to which organizations are motivated to disseminate the guidelines due to a feeling of “ownership” of guideline content and/or material.	“It’s basically what’s funded and what’s not funded, and because you are the guys with all the funding we figure you better do the work because we ain’t got the cash to pay for it.”—Martin, Organization 2 “Generally, we would not do a video if it is not ours.”—Ellen, Organization 7 “I am just really excited to share the work that we have done over the last couple of years”—Lauren, Organization 6	IS

Subthemes were classified using the updated Consolidated Framework for Implementation Research (CFIR) (6). IN, Innovation domain; O, Outer setting domain; IS, Inner setting domain; I, Individuals domain; IM, Implementation process domain.

a Classified as barriers only.

b Classified as facilitators only.

c Classified as barriers and facilitators.

## Discussion

This study offers an original qualitative exploration into the barriers and facilitators to disseminating national movement behavior guidelines experienced by intermediary organizations. Analysis identified four main themes that have the potential to impact the dissemination of guidelines at an organizational level, these include *Compatibility*, *Capacity*, *Actions*, and *Conditions*. Each theme related to at least two CFIR domains, suggesting that intermediary organizations may need support at multiple levels to effectively disseminate national movement behavior guidelines to improve their impact on practice and policy.

Despite the acknowledged importance of guideline dissemination, participants frequently described experiencing constraints to the quantity and quality of guideline dissemination that could be performed. A lack of resources (i.e., financial, human, and materials), the presence of competing priorities, and a low level of decision-making authority were perceived as primary barriers to guideline dissemination. Notably, capacity-related concerns were present regardless of organizational structure or setting. Although some organizational barriers may be difficult to modify (i.e., decision-making authority), these findings suggest that ‘capacity-building’ [i.e., targeting the general capability of an organization to execute an action (6)] and ‘capacity-developing’ [i.e., strengthening existing capabilities of an organization to execute an action (24)] strategies may be a useful avenue for guideline initiatives to support the meaningful and sustained dissemination of national movement behavior guidelines. For example, guideline initiatives may benefit from allocating financial resources to the professional development or training of representatives belonging to intermediary organizations to build confidence in mobilizing guidelines into practice (25). Further, communications toolkits containing scientific guideline documents or public-facing promotional materials tailored to various dissemination settings and target audiences may be developed to ensure the consistent messaging of the guidelines and reduce the burden for material creation (6, 25). Alternatively, organization representatives may benefit from collaborating with a knowledge mobilization specialist to identify existing organization initiatives that may be leveraged or reconfigured to enhance organizational capacity for guideline dissemination. Such an approach aligns with complexity-informed perspectives of knowledge mobilization (26). It may not be feasible for guideline initiatives to pursue the full range of strategies described above, as the knowledge mobilization of national movement behavior guidelines is often under-resourced (10). Further, future research is needed to determine the effectiveness of such strategies for capacity-building and/or development. However, findings suggest that supplementing traditional dissemination approaches with evidence-informed, capacity-focused strategies may enhance the dissemination of national movement behavior guidelines at an organizational level.

Next, participants felt that the alignment of guideline dissemination with an organization’s purpose and/or mission was highly important for dissemination. Indeed, this form of alignment was described as proportional to an organization’s commitment to—and investment in—guideline dissemination. Although movement behavior guidelines are relevant for many health-promoting organizations (10), few organizations exist for the exclusive purpose of promoting healthy movement behavior to the general public. Accordingly, it is challenging for guideline initiatives and knowledge mobilization teams to selectively engage organizations with a high alignment with national movement behavior guideline dissemination. A possible explanation for the poor alignment of guideline dissemination with the mission of many intermediary organizations may stem from the resurrection and rebranding of ParticipACTION in 2007 (27). ParticipACTION is a not-for-profit organization that champions the promotion of healthy movement behavior to the general public (27). It is possible that intermediary organizations do not feel the need to disseminate and institutionalize national movement behavior guidelines as part of their mission or mandate, as this gap is addressed at a national level through the presence of ParticipACTION. However, it is well known that changing movement behavior at a population level necessitates an intersectoral approach, as it is a function of individual behavior, as well as social, environmental, and system influences (28, 29). Accordingly, collaborative and coordinated approaches to guideline knowledge mobilization at multiple levels are needed. Although modifying organizational alignment is beyond the scope of many guideline initiatives, it is possible that guideline initiatives may obtain a greater degree of commitment to—and investment in—guideline dissemination by using a self-nomination process to participate in the development and knowledge mobilization of national movement behavior guidelines.

Lastly, an unanticipated finding was that several participants reported feeling a lack of ownership of the 24HMG, viewing them largely as externally developed resources that they would ‘push’ to their professional network. Indeed, despite using a collaborative, integrated knowledge mobilization approach to inform the knowledge mobilization of the 24HMG (16), several representatives did not report a sense of ownership over guideline materials or messages. This perspective on ownership may stem from the historical dominance of linear approaches to guideline dissemination, where two main sets of actors span the knowledge-to-practice gap [i.e., those that produce knowledge and those that use knowledge (30)]. More recently, researchers are emphasizing the dynamic, multi-faceted, and non-linear nature of knowledge mobilization, suggesting that the process of knowledge mobilization is dependent on social relationships and a shared understanding of knowledge benefits (26). Accordingly, it is necessary for representatives to shift from viewing themselves as passive recipients of guideline documents to active participants in the development and knowledge mobilization of the guidelines (24). In addition to using an integrated knowledge mobilization approach, it is possible that increased ownership of the guidelines may be garnered through using a more collaborative approach to guideline messaging (e.g., expressing a willingness for intermediary organizations to adapt guideline materials and messages for their target audiences, allowing for guideline materials to be co-branded with the logos of intermediary organizations).

### Strengths and limitations

To our knowledge, the salience of the multi-level barriers and facilitators that may influence the dissemination of movement behavior guidelines among intermediary organizations has not been explored globally. This paper makes an important contribution to literature as it represents a starting point for understanding ‘why’ certain barriers or facilitators have the potential to impact the dissemination of movement behavior guidelines in Canada. However, future research is needed to better articulate the specific mechanisms for ‘how’ the determinants identified within this study lead to ‘successful’ or ‘unsuccessful’ guideline dissemination and dissemination outcomes (31). Additional research is also needed to assess whether the determinants of guideline dissemination by intermediary organizations identified in this study are similar in other countries, particularly those whose movement guidelines are embedded as part of policy (32). With this knowledge, we will be better positioned to design interventions to effectively address the challenges to guideline dissemination experienced by intermediary organizations in Canada and beyond. Next, the methodological rigor that was used to explore our research question is a second strength of this work (Supplementary material S4). A collaborative approach was used to design a methodologically coherent study to identify, describe, and understand the barriers and facilitators to national movement behavior guideline dissemination among intermediary organizations in Canada. Although a small convenience sample of representatives participated in this study, the steps taken throughout data analysis ensure that our interpretation of participant perspectives can be viewed as credible and trustworthy.

Despite the strengths of this work, there are some important limitations to consider. First, we used a convenience sample of participants who were involved in the development or knowledge mobilization of the 24HMG to explore our research question. This may have resulted in more similar views or perspectives regarding the barriers and facilitators to guideline dissemination to be represented in our findings. Similarly, although our research team made efforts to engage individuals who held communications or knowledge mobilization roles within their organization, a small number of participants were not responsible for leading knowledge mobilization initiatives at an organizational level. As a result, it is possible that the barriers and facilitators that were described do not fully capture the range of potential barriers and facilitators to guideline dissemination experienced by intermediary organizations in Canada. Further, it is important to acknowledge that this research was completed in June 2020 prior to the release of the 24HMG. Accordingly, it is possible that additional insights may have been generated if interviews had been conducted post-guideline release. Lastly, although some barriers and facilitators were more frequently described by participants positioned in certain dissemination contexts (e.g., education sector versus not-for-profit sector), this study did not explore the relative impact of organizational infrastructure and setting on determinants to guideline dissemination. Future research is needed to understand how organizational characteristics influence guideline dissemination and its outcomes.

## Conclusion

Intermediary organizations play an important role in the knowledge mobilization of national movement behavior guidelines. This study provides insight into the breadth of barriers and facilitators to guideline dissemination that may be experienced by intermediary organizations. Practically, situating our findings within the CFIR allows for guideline development teams and public health practitioners to design interventions to improve the dissemination and impact of national movement behavior guidelines.

## Data Availability

The raw data supporting the conclusions of this article will be made available by the authors, without undue reservation.

## Footnotes

^1^Interviews were completed prior to the release of the 24HMG. As such, the factors that were identified as having an influence on dissemination outcomes were anticipated barriers and facilitators (e.g., they were informed by previous dissemination experiences).

^2^Interview guides were informed by the original Consolidated Framework for Implementation Research (17) as the updated version was unavailable at the time of data collection (June 2020).
