# Supplementary material for: Barriers and facilitators to the dissemination of national movement behavior guidelines among health-promoting organizations: a qualitative study
Source: Front Public Health. 2024 Dec 4;12:1470050. doi: 10.3389/fpubh.2024.1470050 (PMC11652656; doi:10.3389/fpubh.2024.1470050)
Supplement: Supplementary file 1 [file Data_Sheet_1.DOCX]

**Survey items**

**Participant characteristics**

1. What organization do you work for?
2. How long have you worked for this organization? (mm/yy)
3. What is your position within this organization (i.e., the title of your role)?
4. Approximately how many people are there in your organization who… work full time?

….part time or casual?

….volunteer their time?

- - 0
  - <10
  - 10-19
  - 20-29
  - 30-39
  - 40+
  - Not applicable

**Organization characteristics**

1. For how many years has your organization been involved in the promotion of physical activity, sedentary and/or sleep behaviors?
   - <5 years
   - 5-10 years
   - 11-15 years
   - 16-20 years
   - 20+ years
2. Which behavior does your organization primarily promote (i.e., physical activity, sleep, sedentary behavior)? Please select all that apply below.

- Physical activity
- Sedentary behavior
- Sleep

1. Approximately how much of your organization’s work is directly related to the behaviors selected in the previous question?
   - <20%
   - 20-39%
   - 40-59%
   - 60-79%
   - 80-100%
   - Not applicable
2. Does your organization promote any secondary behaviors? Please select all that apply below.

- Physical Activity
- Sedentary behavior
- Sleep
- Other (please specify)

1. Does your organization work mainly at the National, Provincial/Territorial, or Local level?
   - National
   - Provincial/Territorial
   - Local
   - Other (please specify)
2. In which Province or Territory is your organization mainly based?
   - Yukon
   - Northwest Territories
   - Nunavut
   - British Columbia
   - Alberta
   - Saskatchewan
   - Manitoba
   - Ontario
   - Quebec
   - Newfoundland and Labrador
   - New Brunswick
   - Nova Scotia
   - Prince Edward Island
3. Is your organization part of the government, not-for-profit, private, or education sector?
   - Government
   - Not-for-profit
   - Private
   - Education
   - Other (please specify)
4. Has your organization previously disseminated national-level guidelines pertaining to … physical activity?

sedentary behavior?

and/or sleep?

- Yes
- No
