# Supplementary material for: Barriers and facilitators to the dissemination of national movement behavior guidelines among health-promoting organizations: a qualitative study
Source: Front Public Health. 2024 Dec 4;12:1470050. doi: 10.3389/fpubh.2024.1470050 (PMC11652656; doi:10.3389/fpubh.2024.1470050)
Supplement: Supplementary file 2 [file Data_Sheet_2.DOCX]

**Interview guide**

***Introductory statement***

Thank you for taking the time to discuss the dissemination of the upcoming 24 Hour Movement Guidelines for Adults Aged 18-64 Years and Adults Aged 65+ Years. Do you have any questions about the study before we begin?

Do I have your consent to do the interview and begin recording?

***Organization background***

1. Can you tell me a little bit about the mission of your organization?
2. How did your organization become involved in the 24-Hour Movement Guideline knowledge mobilization process?
3. Why do you think your organization has been selected to be a part of the team that is disseminating the 24-Hour Movement Guidelines?
4. What influenced your (your organization's) decision to get involved in the dissemination of the guidelines?
5. Who within your organization will lead guideline dissemination activities? What is their role in your organization?

***Individual characteristics***

1. What do you think of the upcoming 24-Hour Movement Guidelines?
2. How does the guideline align with your organization's values?
3. How do these new guidelines compare to other guidelines that you (or your organization) have had experience disseminating or seen?
4. How confident are you that your organization will be able to successfully disseminate the guidelines?
   1. Why?
5. What effect do you think disseminating the 24-Hour Movement Guidelines will have on the individuals or organizations in your network?

***Guideline perceptions and audience***

1. Think about the individuals and organizations in your communication network. Who will you be disseminating the guidelines to?
   1. What do you think your audience (i.e., the organizations or individuals you are connected with) will think of the upcoming 24-Hour Movement Guidelines?
2. How essential is the guideline to meet the needs of organizations/individuals served by your organization?
   1. How might the dissemination materials (i.e., scientific/public facing document) need to be tailored or refined to meet your organization’s needs?
   2. What kind of supporting evidence or materials are needed to get your audience (i.e., organizations or individuals you are connected to) to buy into the new guidelines?

***Internal influencers of dissemination***

1. Think about the age, maturity, and size of your organization, how might the infrastructure of your organization influence your ability to disseminate guidelines?
   1. To what extent are new ideas embraced and used to make improvements in your organization?
   2. How do you think your organization's culture (general beliefs, values, assumptions that people embrace) will affect the dissemination of the guidelines?
   3. How does your organization typically find out about new information, such as new initiatives, accomplishments, issues in your field?
2. Who are the individuals or groups within your organization that you would like to get on board with guideline dissemination?

***External influencers of dissemination***

1. Think about the other individuals or organizations that your organization is connected to. Which individuals/organizations influence your dissemination activities?
2. What types of pressures does your organization face to be involved with dissemination of the guidelines?
   1. To what extent would disseminating the guidelines provide an advantage for your organization compared to other organizations in your area?
3. Who within your communication network will make the best messenger for dissemination and why?

***Dissemination barriers and facilitators (competing priorities, resources, etc.)***

1. What kinds of dissemination activities has your organization planned for disseminating the upcoming 24-Hour Movement Guidelines?
   1. Why did you pick those strategies?
2. What barriers does your organization face to disseminating the 24-Hour Movement Guidelines?
   1. To what extent might dissemination take a backseat to other high-priority initiatives going on now?
   2. What costs will be incurred by your organization to disseminate the guideline (financial, time, and human resource costs)?
3. What enables your organization to disseminate the 24-Hour Movement Guidelines?
   1. What supports, such as online resources, marketing materials, or a toolkit, are available to help you disseminate the guideline?
   2. How will the available materials effect dissemination in your setting?
4. What does your organization need from the guideline group to ensure successful dissemination?
   1. What additional resources would you require to disseminate the guidelines?

***Evaluation of dissemination activities***

1. Think back to the dissemination activities you mentioned previously, how might these activities/strategies be evaluated to understand whether dissemination was successful?

***Concluding statement***

Thank you for taking the time to speak with me about the dissemination of the upcoming 24-Hour Movement Guidelines

1. Would you like to add any other information related to our interview?
2. Do you have any comments or questions for me?
