# Supplementary material for: Barriers and facilitators to the dissemination of national movement behavior guidelines among health-promoting organizations: a qualitative study
Source: Front Public Health. 2024 Dec 4;12:1470050. doi: 10.3389/fpubh.2024.1470050 (PMC11652656; doi:10.3389/fpubh.2024.1470050)
Supplement: Supplementary file 3 [file Data_Sheet_3.DOCX]

**Pre-interview materials**

Guidelines are useful for setting behavioral benchmarks, developing public health messages, and informing population-level monitoring and surveillance (Brawley and Latimer 2007). However, the creation and release of guidelines, in isolation, are unlikely to facilitate changes in behavior at a population level. Knowledge mobilization strategies, such as dissemination and implementation efforts, are required to increase guideline awareness and adherence among end-users. Dissemination refers to the purposive distribution of a guideline to specific audiences (Rabin et al., 2008), with an aim to enhance guideline awareness, attitudes, and knowledge (Leeman et al., 2015).

A summary of evidence pertaining to the dissemination and evaluation of national physical activity, sedentary behavior, or sleep guidelines is presented below. Please read through these examples and reflect on some dissemination strategies that your organization might employ to disseminate the upcoming 24 Hour Movement Guidelines for Adults Aged 18-64 Years and Adults Aged 65+ Years.

**Definitions of Dissemination**

***Passive Dissemination***: The passive or uncontrolled spread of information or evidence (Brownson, Colditz, & Proctor, 2017).

***Active Dissemination***: Active efforts to spread information using specific strategies or channels (Brownson, Colditz, & Proctor, 2017).

**Examples of dissemination strategies used for previous movement guidelines and how they have been evaluated**

| **Dissemination strategy** | **Dissemination example** | **Evaluation method** |
| --- | --- | --- |
| Distribution of guideline materials | - Scientific statement - Public-facing statement - Print resources developed for target audience (brochures, factsheets, technical reports) - Interactive website of resources | - Cross-sectional survey - Number of ‘hits’ on website using google analytics |
| Mass media/communications campaign | - Website, tv, radio, videos, digital social media, direct marketing | - Number of ‘hits’ on website using google analytics |
| Education/training | - Educational workshops held for target audience to increase awareness of the guidelines | - Cross-sectional survey |
| Dissemination toolkit | - Resources and tools developed to help organizations promote materials to their networks | - Not evaluated |

*Thank you for taking the time to reflect on how your organization might assist in the dissemination of the 24 Hour Movement Guidelines for Adults Aged 18-64 Years and Adults Aged 65+ Years. We look forward to discussing these ideas with you further.*
