# Supplementary material for: Barriers and facilitators to the dissemination of national movement behavior guidelines among health-promoting organizations: a qualitative study
Source: Front Public Health. 2024 Dec 4;12:1470050. doi: 10.3389/fpubh.2024.1470050 (PMC11652656; doi:10.3389/fpubh.2024.1470050)
Supplement: Supplementary file 4 [file Data_Sheet_4.DOCX]

**Consolidated criteria for reporting qualitative studies (COREQ): 32-item checklist**

| **Item No** | | **Description** | **Reported on Page #** |  |
| --- | --- | --- | --- | --- |
| **Domain 1: Research team and reflexivity** | | | |  |
| **Personal Characteristics** | | | |  |
| 1. Interviewer/ facilitator | | First author | Page 3 |  |
| 2. Credentials | | PhD | Page 2 |  |
| 3. Occupation | | Doctoral student | Page 2 |  |
| 4. Gender | | Female | Page 3 |  |
| 5. Experience and training | | The first author was a doctoral student in the field of health promotion at the time of data collection with training in implementation science. | Page 2 |  |
| **Relationship with participants** | | | |  |
| 6. Relationship established | | The first author had some familiarity with study participants through their involvement on the Knowledge Mobilization Advisory Committee for the Canadian 24-Hour Movement Guidelines. | Page 2-3 |  |
| 7. Participant knowledge of the interviewer | | Participants had some familiarity with the first author, as noted above, and all participants were aware of the objective of the research (i.e., to explore the barriers and facilitators that intermediary organizations experience to national movement behavior guideline dissemination). | Page 2 |  |
| 8. Interviewer characteristics | | The above characteristics of the first author are described in our positionality statement. The first author recognizes that their background may have influenced data collection and analysis in various ways. For example, it is possible that their theoretical background and knowledge of implementation science shaped the organizational barriers and facilitators that were described in this study. In addition, their access to study participants was aided by their previous experience completing formative research for the 24HMG. | Page 2-3 |  |
| **Domain 2: study design** | | |  |  |
| **Theoretical framework** | | |  |  |
| 9. Methodological orientation and Theory | We approached our work from a critical realist paradigm and used inductive thematic analysis to generate themes. | Page 2 |  |  |
| **Participant selection** | | |  |  |
| 10. Sampling | A convenience sampling method was used to select participants in this study. | Page 3 |  |  |
| 11. Method of approach | Participants were invited to participate via email. | Page 3 |  |  |
| 12. Sample size | 16 participants representing 14 organizations participated in this study. | Page 3 |  |  |
| 13. Non-participation Setting | Not applicable |  |  |  |
| 14. Setting of data collection | Data was collected virtually using Zoom. | Page 3 |  |  |
| 15. Presence of nonparticipants | Not applicable |  |  |  |
| 16. Description of sample | Organization characteristics are detailed in Table 1. | Page 4 |  |  |
| **Data collection** | | |  | No |
| 17. Interview guide | The interview guide was structured using the Consolidated Framework for Implementation Research and pilot tested prior to use. | Page 3 |  |  |
| 18. Repeat interviews | Not applicable |  |  |  |
| 19. Audio/visual recording | All interviews were audio recorded. | Page 3 |  |  |
| 20. Field notes | Field notes were kept by the first author throughout the interview process. | Page 3 |  |  |
| 21. Duration | Interviews ranged from 38-69 minutes in duration. | Page 3 |  |  |
| 22. Data saturation | Not applicable |  |  |  |
| 23. Transcripts returned | Transcripts were not returned to participants following interviews. |  |  |  |
| **Domain 3: analysis and findings** | | |  |  |
| **Data analysis** | | |  |  |
| 24. Number of data coders | The first author coded participant interviews and developed the resulting themes. A research assistant and a critical friend were also engaged throughout analysis to encourage reflexivity. | Page 3 |  |  |
| 25. Description of the coding tree | A description of each theme and subtheme can be found in Table 2. | Page 10 |  |  |
| 26. Derivation of themes | Each theme was developed inductively from the data. | Page 3 |  |  |
| 27. Software | NVivo Version 12 was used throughout analysis. | Page 3 |  |  |
| 28. Participant checking | Member checking was not used within this study. |  |  |  |
| **Reporting** | | |  |  |
| 29. Quotations presented | Participant quotations are used to illustrate the themes identified in this study. Pseudonyms are used to protect participant confidentiality. | Page 5-9 |  |  |
| 30. Data and findings consistent | There is consistency between the data presented and the discussion of study findings. | Page 5-9 |  |  |
| 31. Clarity of major themes | All themes are clearly and richly depicted in the results section. A description of each theme and subtheme can be found in Table 2. | Page 5-9 |  |  |
| 32. Clarity of minor themes | Adjectives are used throughout the manuscript to demonstrate the degree of alignment amongst participants for each subtheme described (e.g., a number of participants, a few participants, all participants, etc.). When applicable, contrasting views or singular views are also highlighted. | Page 5-9 |  |  |
